# Hypnic headache: a rare type of primary headache disorder

**DOI:** 10.1192/bjo.2021.349

**Published:** 2021-06-18

**Authors:** Muhammad Sayed Inam, Saifun Nahar, Mohammad Zubayer Miah

**Affiliations:** 1 Sylhet MAG Osmani Medical College and Hospital; 2 National Institute of Mental Health Dhaka; 3 Shaheed Tajuddin Ahmed Medical College

## Abstract

**Objective:**

Hypnic Headache are is a very rare primary headaches that affect the elderly, with an average age of 60 years. Research in the areas of neurophysiology and treatment options for Hypnic Headache are necessary in order to better understand, and improve outcomes for this rare headache disorder.

**Case report:**

Mr. X is a 70-year-old patient, has been presenting with the complaints of headache during sleep at night for the last 1 year. The Headache started after 3 to 4 hours after falling asleep. Due to headache, he wakes up from sleep around 03:00 to 04:00 am almost every night and his headache persist for 30 to 40 minutes. After waking up from sleep he keeps himself busy with religious activity and the headache gradually resolves. He then goes back to bed again.

Mr. X also informed that, the headache is dull in nature and located in left temporo occipital region. It is not associated with photophobia, phonophobia, nausea, vomiting, tearing or discomfort in the leg. He gives no history of early morning headache or day time headache, sleep disorder, snoring or sleep apnea. He has no past history of trauma to the head, fainting attack, unconsciousness, weakness or paralysis of limbs, seizures or non-epileptic seizures. He is an non-smoker, non-alcoholic, non-hypertensive & non-diabetic person.

On general examination, his heart rate is 70 beats/min, blood pressure 138/68 mm of Hg. There are no anemia, jaundice or oedema present in him. His both lung fields are clear. On neurological examinations there are nothing abnormality detected. His Serological investigations, CBC (Complete Blood Count) FBS (Fasting Blood Glucose), lipid profile are within normal limit. CT scan of the brain is normal. There are no cerebral atrophy or volume loss compatible with age.

Mr. X was treated by several general practitioners with paracetamol, diclofenac sodium, mefenamic acid, tramadol hydrochloride. He used these drugs either singly or in combinations. But with this treatment there were no significant improvement occurs. Mr. X is scared and depressed for his sleep time headache.

**Discussion:**

Hypnic headache is a very rare headache disorder. It occurs in age groups over 60 years. It is occur at night during in sleep and waking the patient up, hence the name of it “alarm clock headache”. It is commonly unilateral and lasts for 15 minutes to 4 hours. Hypnic headache commonly dull or throbbing in character and does not make the patient restless, unlike in Cluster Headache. After waking up from sleep, most patients engage in some activity. Hypnic headache is not associated with rhinorrhea, tearing and ptosis. Diagnosis is mainly clinical. Secondary causes headache must be excluded. International Classification of Headache Disorders 3rd Edition (ICHD-3)-beta provides diagnostic criteria for hypnic headache. Pathophysiology of hypnic headache is not clearly identified. Usual treatment options of Hypnic headache includes bed time coffee, lithium carbonate, indomethacin. Our patient fulfil all the criteria of Hypnic headache and he fells improvement with Indomethacin 50 mg in devided doses.

**Conclusion:**

Hypnic Headache is a very rare type of primary headache. It should be diagnosed only after other secondary causes of headache have been excluded. Caffeine, lithium carbonate, flunarizine, indomethacin, used to treat the patient of Hypnic Headache. Lack of study and awareness about these disorders can lead to delays in diagnosis and treatment. Clinical trials are needed to find out proper treatment, but it will be difficult to perform because of the rareness of this disorder.

